# Variations in the Physical Properties of RF-Sputtered CdS Thin Films Observed at Substrate Temperatures Ranging from 25 °C to 500 °C

**DOI:** 10.3390/nano12101618

**Published:** 2022-05-10

**Authors:** Sangwoon Lee, Juna Kim, Seokhee Lee, Hyun-Jin Cha, Chang-Sik Son, Young-Guk Son, Donghyun Hwang

**Affiliations:** 1 School of Materials Science and Engineering, Pusan National University, Busan 46241, Korea; 201983325@pusan.ac.kr (S.L.); kja6037@pusan.ac.kr (J.K.); leesh91@pusan.ac.kr (S.L.); jk260df@pusan.ac.kr (H.-J.C.); 2 Division of Materials Science and Engineering, Silla University, Busan 46958, Korea; csson@silla.ac.kr

**Keywords:** cadmium sulfide, thin film, substrate temperature, RF magnetron sputtering, physical property

## Abstract

CdS films with a wide range of substrate temperatures as deposition parameters were fabricated on Corning Eagle 2000 glass substrates using RF magnetron sputtering. The crystallographic structure, microscopic surface texture, and stoichiometric and optical properties of each CdS film deposited at various substrate temperatures were observed to be highly temperature-dependent. The grown CdS thin films revealed a polycrystalline structure in which a cubic phase was mixed based on a hexagonal wurtzite phase. The relative intensity of the H(002)/C(111) peak, which represents the direction of the preferential growth plane, enhanced as the temperatures climbed from 25 °C to 350 °C. On the contrary, the intensity of the main growth peak at the higher temperatures of 450 °C and 500 °C was significantly reduced and exhibited amorphous-like behavior. The sharp absorption edge revealed in the transmission spectrum shifted from the long wavelength to the short wavelength region with the rise in the substrate temperature. The bandgap showed a tendency to widen from 2.38 eV to 2.97 eV when the temperatures increased from 25 °C to 350 °C. The CdS films grown at the temperatures of 450 °C and 500 °C exhibited glass-like transmittance with almost no interference fringes of light, which resulted in wide bandgap values of 3.09 eV and 4.19 eV, respectively.

## 1. Introduction

Cadmium sulfide (CdS) is a “Group II-VI compound semiconductor formed by cadmium (Cd) belonging to Group IIB metallic elements and sulfur (S) included in Group VI non-metallic elements” [1]. CdS is one of the *n*-type semiconductor materials exhibiting a bandgap of 2.42 eV [2]. Due to these characteristics, it is mainly applied to optoelectronic devices such as photovoltaics [3,4]. Moreover, several researchers are focusing on CdS because it can be applied to devices, such as piezoelectric, photonic, and thin-film transistors [5,6,7]. CdS thin films are deposited using various techniques, such as “sputtering” [8], “molecular beam epitaxy (MBE)” [9], “chemical vapor deposition (CVD)” [10], “chemical bath deposition (CBD)” [11], and “spray pyrolysis” [12]. Each of these deposition techniques has its own set of advantages and disadvantages. One of the widely utilized deposition process techniques for fabricating CdS films more efficiently is radio-frequency (RF) magnetron sputtering. RF magnetron sputtering enables better adhesion, wider coverage, higher uniformity, the convenience of thickness control, and higher film density at relatively low substrate temperatures in the film manufacturing process compared to other deposition techniques [8]. The physical characteristics of RF magnetron sputtered CdS thin films are directly dependent on deposition parameters, such as “the substrate temperature, working pressure, gas flow rate, growth time, and RF power” [8,13,14]. Among these deposition parameters, “the substrate temperature is the main experimental factor that determines the crystallographic, optical and electrical properties of thin films” [13,14]. The surface texture and thickness of the grown thin film also change significantly corresponding to the marginal variation in the substrate temperature during the sputtering process. Moreover, the desired phase can be obtained if the substrate temperature is sufficiently high despite insufficient sputtering power or deposition pressure. N. Akcay et al. “fabricated CdS thin films with the thickness of 50 nm or less at the substrate temperature of 200 °C using RF magnetron sputtering and applied it as the buffer layer for CZTS thin-film solar cells” [13]. Das et al. “demonstrated the effect of the temperatures on various properties of RF-sputtered CdS thin films grown at substrate temperatures ranging from 25 °C to 300 °C” [14]. In a previous study, we also discussed “the structural and optical properties of CdS thin films prepared at the temperatures between 25 °C and 250 °C” [15]. Most studies on the application of CdS films as buffer layers explain the physical properties at substrate temperatures below 300 °C. However, the characteristics of the devices may be determined based on the characteristics of CdS films exhibited at substrate temperatures above 300 °C when the CdS film is applied as a junction layer for electronic devices other than the buffer layer of solar cells [16,17,18]. Therefore, further research on the physical properties of CdS thin films over a wide range of substrate temperatures is required.

In this paper, CdS thin films were fabricated on glass substrates by RF magnetron sputtering by applying a wider range of substrate temperatures (25–500 °C) as experimental parameters than in previous studies. In particular, the singularity of structural and optical property fluctuations seen at 450 °C and 500 °C is a unique result that has not been previously reported in other papers. Additionally, we discussed in depth how deposition temperatures affect the structural, morphological, compositional, and optical aspects of CdS thin films.

## 2. Materials and Methods

### 2.1. Deposition of CdS Thin Films

CdS thin films with various substrate temperatures were prepared on Corning Eagle 2000 glass substrates by RF magnetron sputtering. Corning Eagle 2000 glass substrate features a strain point of 666 °C and a softening point of 985 °C and has better thermal stability than soda-lime glass, which has a strain point of 520 °C and a softening point of 820 °C. The CdS target used in the sputtering process was 4 mm thick, 50 mm in diameter, and 99.99% pure (4N). A 25 × 25 mm^2^ glass was used as the substrate for CdS thin-film deposition, and the fine glass particles generated during glass cutting were blown away using a nitrogen gun. Glass substrates were placed in a beaker containing deionized (DI) water and ultrasonically cleaned for 10 min to remove residual organic substances on the surface of the substrate. The substrates were immersed in a beaker containing a 99% or higher purity of acetone, ethyl alcohol, and isopropyl alcohol, followed by ultrasonic cleaning for 5 min after each post-primary cleaning. The surfaces of the substrates after ultrasonic cleaning were dried using a nitrogen gun and immediately loaded into a vacuum chamber to minimize external contamination. The initial vacuum degree of the chamber for the sputtering process was 5.0 × 10^−6^ Torr (0.667 mPa) or less using a turbopump. Moreover, 55 sccm of argon gas was introduced into the chamber through a mass flow controller. The surface impurities of the CdS target were removed by performing pre-sputtering for 30 min at an RF power of 60 W and a deposition pressure of 3.0 × 10^−2^ Torr (4 Pa) before the deposition of a CdS thin film. The substrate was isolated from the target by a movable shutter during the pre-sputtering and cleaning of the target surface. Thereafter, CdS thin films were deposited on the substrates for 15 min by increasing the RF power to 120 W and adjusting the temperature from 25 °C to 500 °C at the same deposition pressure.

### 2.2. Characterization of CdS Thin Films

The crystallographic characteristics of the CdS thin films were analyzed using X-ray diffraction (XRD, D8 Advance, Bruker, Billerica, MA, USA) with a Cu Kα radiation wavelength (λ) of 0.15406 nm. The surface morphology, grain size, film thickness, and elemental composition of the CdS thin films were evaluated by a field emission scanning electron microscope (FESEM, S-4800, HITACHI, Tokyo, Japan) and an energy dispersive X-ray spectrometer (EDS, 7953-H, Horiba, Kyoto, Japan) attached as an additional accessory. The optical transmittance was observed under a wavelength variable of 190 to 1100 nm through an ultraviolet-visible (UV–Vis) spectrometer (UV-1800, SHIMADZU, Kyoto, Japan). The energy bandgap was calculated based on the data collected from the transmittance measurements.

## 3. Results and Discussion

### 3.1. Structural Properties of CdS Thin Films

**Figure 1:**
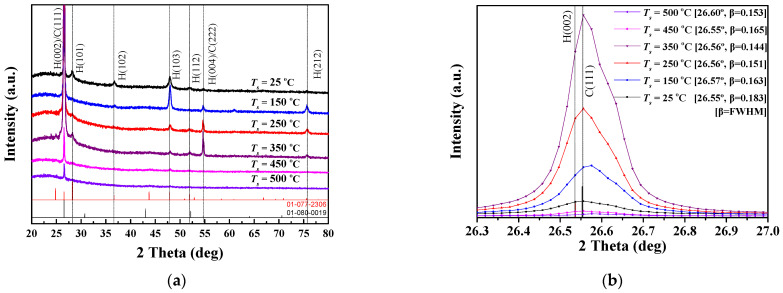
(**a**) XRD results of CdS thin films fabricated at various substrate temperatures, and (**b**) the details of the H(002)/C(111) peaks corresponding to the main diffraction plane in the XRD patterns.

Figure 1 illustrates the XRD patterns of CdS thin films deposited at various substrate temperatures (*T_s_*) measured at 2 theta (*θ*) diffraction angles ranging from 20° to 80°. The deposited CdS films were mixed in hexagonal (H) and cubic (C) structures (JCPDS No. 01-077-2306 and 01-080-0019) [19]. According to the JCPDS card showing information about the CdS crystal system, it is difficult to ascertain a distinct Miller index for the XRD pattern represented at 2θ diffraction angles of 26.5° and 54.6°. This is due to the 2θ angles representing the hexagonal and cubic structures as being 26.53° and 26.55°, respectively, with the difference between the two angles being only 0.02. The angular difference between the hexagonal and cubic structures expressed at 54.64° and 54.67°, respectively, is also very insignificant at 0.03. Therefore, the diffraction peaks appearing at these two angles can be expressed by the Miller indices of H(002)/C(111), which represent the mixture of hexagonal and cubic phases [19,20].

The peaks for the H(002)/C(111) plane representing the preferential growth orientation were observed at a diffraction angle of 26.5° [21,22]. Relatively weak peaks corresponding to the H(101), H(102), H(103), H(112), and H(212) planes representing the hexagonal structure were observed at diffraction angles of 28.2°, 36.6°, 47.8°, 51.9°, and 75.6°, respectively. As the temperature ascended to 350 °C, the intensity of the principal diffraction peaks increased proportionately. However, the intensity of the dominant peaks dropped dramatically for the films grown at higher temperatures (450 °C and 500 °C). The intensity of the peak on the H(004)/C(222) plane at a diffraction angle of 54.6° depended on the substrate temperature at the main peaks. The full width at half maximum (FWHM) of the H(002)/C(111) peaks for CdS thin films deposited at a variety of substrate temperatures is summarized in Table 1. The FWHM values were analyzed using DIFFRAC.SUITE software, which was provided by Bruker. The Scherrer formula (*D =* 0.9*λ/βcosθ*) was used to determine the crystallite size [23]. The crystallite size of the CdS thin film at 25 °C was 46.6 nm. The crystallite size gradually increased to 52.3 nm at 150 °C, 56.4 nm at 250 °C, and 59.2 nm at 350 °C, corresponding to the increase in substrate temperature. However, for the films formed at 450 °C and 500 °C, the crystallite size was reduced to 51.7 nm and 55.7 nm, respectively.

### 3.2. Morphological and Compositional Properties of CdS Thin Films

**Figure 2:**
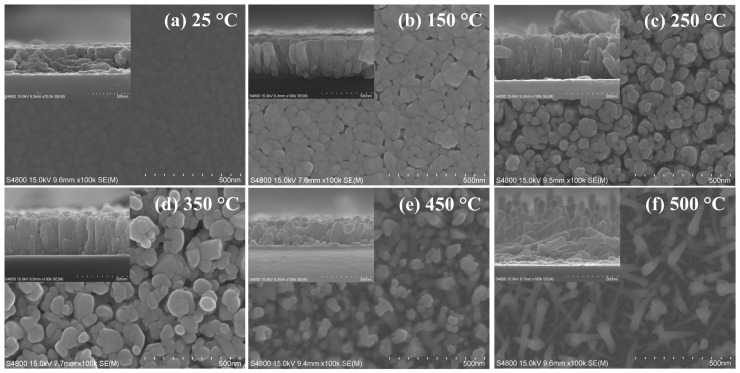
Surface morphology and cross-sectional FESEM images of CdS thin films at (**a**) *T_s_* = 25 °C, (**b**) *T_s_* = 150 °C, (**c**) *T_s_* = 250 °C, (**d**) *T_s_* = 350 °C, (**e**) *T_s_* = 450 °C and (**f**) *T_s_* = 500 °C.

Figure 2 represents the FESEM images of the surface morphology and cross-section of the prepared CdS thin films at various substrate temperatures. The crystal grains with an average diameter of 43.3 nm are closely bonded to each other to form a thin-film surface in the CdS film prepared at the temperature of 25 °C. A temperature of 25 °C corresponds to a relatively low temperature in terms of promoting “initial nucleation and providing a high density of nuclei to obtain a surface texture” [24]. When growing a thin film by sputtering, if the substrate temperature is raised while fixing the deposition time, sufficient activation energy for nucleation and growth is supplied according to the temperature increase during the deposition process. Therefore, the substrate temperature becomes an effective process condition to improve the density and thickness of the thin film and the grain size [25]. The average grain size value for CdS films improved to 77.2 nm when the temperature was elevated to 350 °C. Moreover, the spacing of adjacent grain boundaries also widened. The average thickness of the CdS film deposited at 25 °C was measured to be 333 nm. The thickness of the film grew with increasing temperatures to 378 nm at 150 °C and 437 nm at 250 °C. Through these observations, it could be seen that the thickness of the CdS thin film deposited by the sputtering process gradually improved with the substrate temperature up to 250 °C. The thickness of the films deposited at 350 °C and 450 °C, on the other hand, plummeted to 396 nm and 254 nm, respectively. The decrement in the thickness of the films observed at the temperatures between 350 °C and 450 °C is due to the re-evaporation phenomenon occurring at higher substrate temperatures above 300 °C [24,26]. The surface and cross-sectional images of the CdS thin film deposited at 450 °C show that the crystals grew in extremely irregular directions under these conditions. The crystal growth irregularities were more pronounced in the images of CdS films deposited at the temperature of 500 °C, exhibiting a shape that resembled nanorods. The surface morphology and thickness variations occurring at high temperatures over 450 °C are consistent with the amorphization tendency observed in the XRD results. The structural and optical singularities at 450 °C and 500 °C were observed for the first time during our research.

The results of the EDS analysis of the chemical composition are listed in Table 2. The average values of the four different points are indicated. The atomic percentage of Cd was higher than that of S for CdS films deposited at temperatures between 25 °C and 350 °C. The atomic percentage of Cd represents a continuously decreasing trend with increasing substrate temperature. The reduction of Cd content is presumed to be due to the surface mobility and vapor pressure of the Cd element being higher than that of the S element, so that the Cd atoms preferentially escape from the CdS film along with the rise in the substrate temperature [14]. The atomic ratio of elemental S to Cd in the films grown at the temperatures between 250 °C and 350 °C was approximately equal to the stoichiometric composition of CdS. Conversely, the atomic percentage of S was higher than that of Cd at 450 °C and 500 °C. The S/Cd ratio was also calculated to be 1.14 and 1.09, which are values that deviate from the stoichiometric composition. The results of the EDS investigation show that the deterioration of the crystallinity and the non-uniformity of the surface microstructure of the CdS thin films grown at substrate temperatures of ≥450 °C can be attributed to the collapse of the stoichiometry induced by the volatilization of the Cd element [27,28,29].

### 3.3. Optical Properties of CdS Thin Films

**Figure 3:**
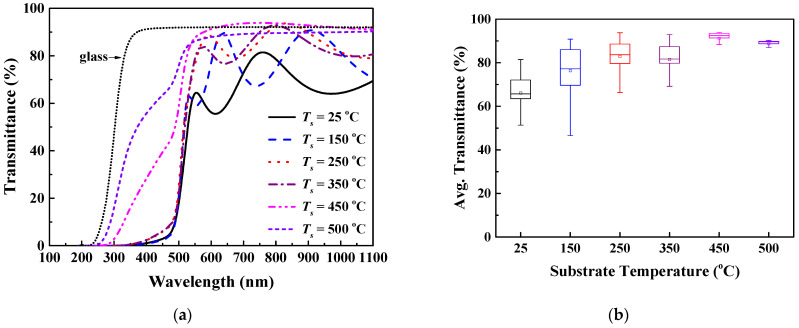
Optical transmittance spectral results for CdS thin films fabricated at various substrate temperatures: (**a**) transmittance spectrum and (**b**) average transmittance distribution.

Figure 3 shows the optical transmittance results for CdS thin films fabricated over a wide substrate temperature range. The sharp absorption edge of the CdS thin film deposited at 350 °C was observed near the wavelength of 500 nm. However, at higher temperatures between 450 °C and 500 °C, the absorption edge wavelengths were confirmed at 300 nm and 270 nm, respectively. The average transmittance of the room-temperature-sputtered CdS thin film measured for wavelengths between 500 to 1100 nm was 66%. The transmittance values for the CdS thin film improved to 76.3% at 150 °C, 82.8% at 250 °C, 81.5% at 350 °C, and 91.2% at 450 °C with the increase in the substrate temperature. At a substrate temperature of 500 °C, the transmittance decreased to 88.8% compared to the immediately preceding condition. The absorption edge of the transmittance spectra at substrate temperatures of 450 °C and 500 °C shifted to the shorter wavelength region. The blue shift of the absorption edge is thought to be related to the shape of the crystal’s growth. We previously mentioned the singularity of the shape variation of the surface and cross-section of CdS films grown at 450 °C and 500 °C through the SEM results in Figure 2. In the deposited films under these conditions, the spacing between adjacent crystal grains is unusually wide. It also indicates that the crystal growth was randomly grown in an irregular direction rather than perpendicular to the substrate. Due to these results, it is thought that the transmittance pattern of the deposited CdS film in this temperature range exhibits an amorphous-like behavior. Moreover, the interference fringes of light observed in the 500 to 1100 nm wavelength range were absent. This may be due to the volatilization of the Cd element, which occurs when exposed to temperatures above 450 °C, as indicated in Table 2.

**Figure 4:**
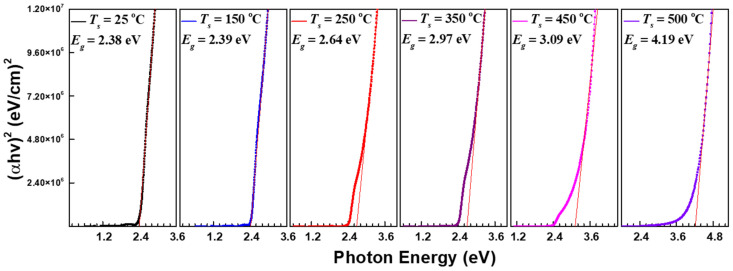
Energy band gap of CdS thin films as determined by transmittance spectroscopy.

The energy bandgap of the CdS thin film was “calculated by substituting the absorption coefficient (*α*) derived from the transmission spectrum into the following Tauc equation” and is shown in Figure 4 [30].

(*αhυ*)^1/*n*^ = *A*(*hυ* − *E_g_*) (1)

In this equation, “*A* is the constant for the effective mass (*M*) associated with the bands, and *h* is Planck’s constant. The frequency of the incident radiation is *υ*, the bandgap of that material is *E_g_*, and the exponent *n* is the transition probability” [14,30]. The transition probability, *n*, was calculated by substituting the value of the direct allowed transition (1/2) in Equation (1). The *E_g_* was determined by approximating the (*αhυ*)^2^ graph on the *y*-axis corresponding to the photon energy (*hυ*) extrapolated to *α* = 0 with a straight line [2,31]. The bandgap of the CdS thin film deposited at a substrate temperature of 25 °C was 2.38 eV. “This bandgap value is higher than the bulk of CdS at 300 K (2.42 eV)” [32]. The bandgap value widened from 2.39 eV to 2.97 eV as the substrate temperature was raised from 150 °C to 350 °C. The analysis of XRD patterns (Figure 1), FESEM images (Figure 2), and EDS data (Table 2) established that substrate temperatures of up to 350 °C were effective deposition parameters for the formation of CdS thin films. Therefore, it can be considered that this temperature range has a positive effect on the grain size, crystal structure, and stoichiometric composition in the growth stage of the CdS thin films. The band gaps at higher substrate temperatures (450 °C and 500 °C) were 3.09 eV and 4.19 eV, respectively. This steep increase in the bandgap is due to the blue shifting of the transmission spectrum following the amorphous nature of the CdS thin films. It should be noted here that the measured bandgap values under these substrate temperature conditions may be somewhat inaccurate. The thickness of the film is one of the important parameters when plotting the bandgap with the Tauc equation. The cross-sectional SEM image of the 450 °C samples shown in Figure 2 represents a higher density than the 500 °C samples. The thickness of the sample at 450 °C was calculated based on the average of the points where the particles grown perpendicular to the substrate were densely distributed. At a substrate temperature of 500 °C, grain growth occurs randomly in a direction perpendicular or oblique to the substrate. Since the length of each grown crystal is different, it is only possible to estimate the film thickness rather than accurately measuring the film thickness. The film thickness of the sample was calculated based on the average length of crystals grown at perpendicular or very close angles. Therefore, depending on which viewpoint is applied, the thickness of the film deposited at 500 °C may differ from the value of 569 nm shown in Table 2; the actual thickness is estimated to be much thinner than that value. For these reasons, the inhomogeneous substrate coverage observed at 450 °C was further aggravated at 500 °C, leading to accelerated amorphous behavior and an increased bandgap [33].

## 4. Conclusions

In this study, we comprehensively analyzed the effect of substrate temperature on the structural, compositional, and optical properties of RF-sputtered CdS thin films. All CdS thin films grown at various substrate temperatures (25–500 °C) showed a polycrystalline structure with mixed hexagonal (H) and cubic (C) phases. The diffraction peak for the H(002)/C(111) plane was observed at 26.5°, indicating a preferential growth orientation. The diffraction intensity for the main peak showed a tendency to significantly improve more than twofold as the substrate temperature increased from 25 °C to 350 °C. In contrast, the intensity for the peak at the temperature conditions of 450 °C and 500 °C decreased and showed a value significantly lower than that of the CdS film deposited at 25 °C. FESEM image analysis confirmed that substrate temperature has a respectable effect on morphological changes such as the surface and thickness of CdS thin films. In particular, in the image of the CdS thin film grown at 500 °C, it was confirmed that crystal growth was performed in an arbitrary direction rather than perpendicular to the substrate. The distance between the grains was also observed to widen compared to the lower substrate temperature, revealing non-uniform crystal growth. The stoichiometric ratio (S/Cd) of the CdS films grown at 250 °C and 350 °C showed the same value of 0.97. This value is slightly less than the stoichiometric ratio of bulk CdS. The volatilization of Cd was observed in CdS films prepared at higher temperatures (450 °C and 500 °C). Therefore, the chemical composition of the films represents an S-rich stoichiometry. The sharp absorption edge of the CdS thin film fabricated at a substrate temperature of 25 °C was observed near a wavelength of 500 nm, and the average transmittance was 66%. Subsequently, the transmittance of the film was enhanced to about 81.5% and 91.2% at temperatures of 350 °C and 450 °C, respectively. At the substrate temperature of 500 °C, the transmittance decreased slightly compared to the previous condition and showed a value of approximately 88.8%. The band gaps of the CdS thin films grown at substrate temperatures of 25 °C and 150 °C were 2.38 eV and 2.39 eV, respectively, which were close to those of bulk CdS. The bandgap of the CdS thin film deposited at the higher substrate temperature presented a tendency to widen in proportion to the increase in temperature.

## Figures and Tables

**Table 1:** Detailed values representing the structural properties of CdS thin films fabricated at various substrate temperatures.

Substrate Temperature (°C)	2 Theta (deg)	FWHM Value (deg)	Crystallite Size by XRD (nm)	Grain Size by FESEM (nm)	Thickness by FESEM (nm)
25	26.55	0.183	46.6	43.3	333
150	26.57	0.163	52.3	49.4	378
250	26.56	0.151	56.4	65.4	437
350	26.56	0.144	59.2	77.2	396
450	26.55	0.165	51.7	47.8	254
500	26.60	0.153	55.7	55.3	569

**Table 2:** Values of the variation in the chemical composition of CdS thin films grown at various substrate temperatures.

Substrate Temperature (°C)	Cd (Atomic %)	S (Atomic %)	S/Cd Ratio
25	53.15	46.85	0.88
150	51.59	48.41	0.94
250	50.89	49.11	0.97
350	50.87	49.13	0.97
450	46.57	53.43	1.14
500	47.76	52.24	1.09

## Data Availability

Not applicable.
